# SDNET2018: An annotated image dataset for non-contact concrete crack detection using deep convolutional neural networks

**DOI:** 10.1016/j.dib.2018.11.015

**Published:** 2018-11-06

**Authors:** Sattar Dorafshan, Robert J. Thomas, Marc Maguire

**Affiliations:** a Department of Civil and Environmental Engineering, Utah State University, Logan, Utah. USA; b Department of Civil and Environmental Engineering, Clarkson University, Potsdam, NY, USA

## Abstract

SDNET2018 is an annotated image dataset for training, validation, and benchmarking of artificial intelligence based crack detection algorithms for concrete. SDNET2018 contains over 56,000 images of cracked and non-cracked concrete bridge decks, walls, and pavements. The dataset includes cracks as narrow as 0.06 mm and as wide as 25 mm. The dataset also includes images with a variety of obstructions, including shadows, surface roughness, scaling, edges, holes, and background debris. SDNET2018 will be useful for the continued development of concrete crack detection algorithms based on deep convolutional neural networks (DCNNs), which are a subject of continued research in the field of structural health monitoring. The authors present benchmark results for crack detection using SDNET2018 and a crack detection algorithm based on the AlexNet DCNN architecture. SDNET2018 is freely available at https://doi.org/10.15142/T3TD19.

**Specifications table**

**Table:** 

Subject area	Structural health monitoring, deep learning, convolutional neural networks, artificial intelligence
More specific subject area	Concrete crack detection, image classification
Type of data	2D-RGB image (.jpg)
How data was acquired	Original images of cracked and non-cracked concrete bridge decks, walls, and pavements were captured using a 16 MP Nikon digital camera.
Data format	Raw digital images (.jpg)
Experimental factors	
Experimental features	• 230 images of cracked and non-cracked concrete (54 bridge decks, 72 walls, 104 pavements) segmented into more than 56,000 sub-images (256 × 256 px) • Crack widths from 0.06 to 25 mm • Obstructions including shadows, surface debris, inclusions, scaling, etc…
Data source location	Utah State University, Logan, Utah, USA
Data accessibility	The dataset is freely accessible at [1] for any academic purposes
Related research article	Parts of this dataset have been used in the following research items for image-based non-contact crack detection applications: [2], [3], [4], [5], [6], [7], [8]

**Value of the data**

• SDNET2018 can be used for training, validation, and benchmarking of algorithms for autonomous crack detection in concrete;
• SDNET2018 has images of reinforced concrete decks (D) and walls (W), and unreinforced concrete pavements (P), which enables DCNNs training on it while also categorizing different types of concrete cracks;
• A DCNN trained on SDNET2018 can identify fine and wide cracks due to the size variety in it, widths from 0.06 mm to 25 mm;
• Images in SDNET2018 intentionally include irrelevant objects which may improve the accuracy of DCNNs trained on this dataset in real applications;
• SDNET2018 can be used to develop new DCNN architectures or modify the existing architectures, e.g. AlexNet or GoogleNet, in order to increase the efficiency of the network for concrete crack detection.

## 1 Data

The SDNET2018 image dataset contains more than 56,000 annotated images of cracked and non-cracked concrete, bridge decks, walls, and pavements. Its purpose is for training, validation, and benchmarking of autonomous crack detection algorithms based on image processing, deep convolutional neural networks (DCNN) [8], or other techniques. Such techniques are increasing in popularity in the structural health monitoring field. Continued advancement of crack detection algorithms requires an annotated diverse image dataset [9], which has not been available until now.

**Fig. 1:**
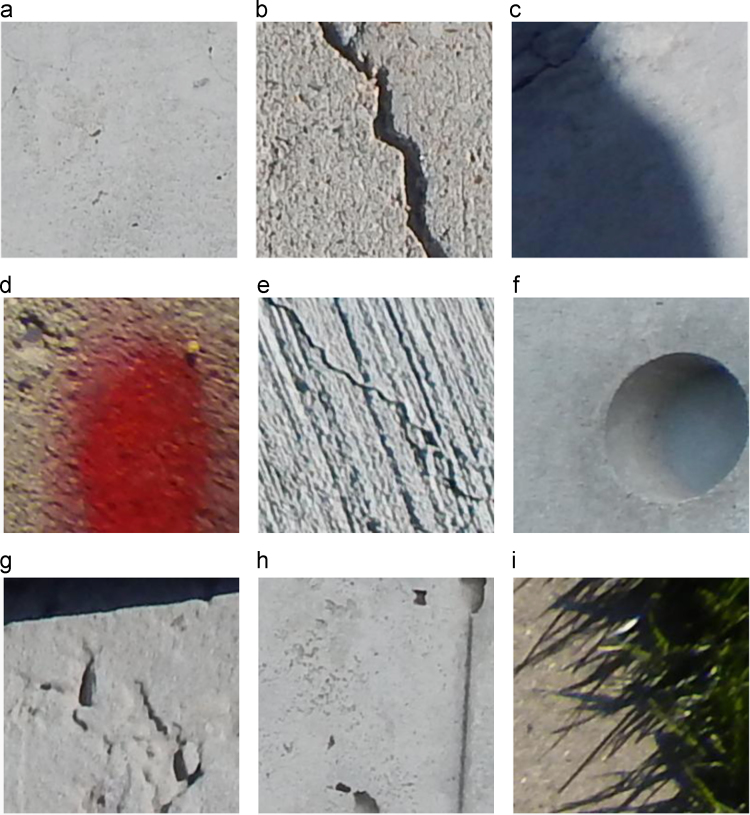
SDNET2018 images include (a) fine cracks, (b) coarse cracks, (c) shadows, (d) stains, (e) rough surface finishes, (f) inclusions and voids, (g) edges, (h) joints and surface scaling, and (i) background obstructions.

Images of bridge decks were taken at the Systems, Materials, and Structural Health (SMASH) Laboratory at Utah State University, where a number of full scale bridge deck sections were stored. Images of walls and pavements were taken on Utah State University campus. Table 1 lists the number of cracked, non-cracked, and total sub-images of each type included in SDNET2018. The sample images in Fig. 1 show the range of crack widths, surface conditions, and other environmental factors represented within SDNET2018. Images are 256 × 256-px RGB image files in .jpg format. Each image is classified as cracked or non-cracked and stored in a corresponding folder within the repository. Images are organized into three sub-directories: P for pavements, W for walls, and D for bridge decks. Each subfolder is further organized into sub-sub-directories with the prefix C for cracked and U for uncracked (e.g.,:/D/CD for images of bridge decks with cracks). With the exception of segmentation into sub-images as discussed above, the images have not been modified from their original state.

**Table 1:** SDNET2018 image dataset description and statistics.

**Image description**		**No. cracked**	**No. non-cracked**	**Total**
Reinforced	Bridge deck	2025	11,595	13,620
	Wall	3851	14,287	18,138
Unreinforced	Pavement	2608	21,726	24,334
Total		8484	47,608	56,092

## 2 Experimental design, materials, and methods

SDNET2018 images were taken with a 16-MP Nikon camera at a working distance of 500 mm without zoom. The sensitivity was 125 ISO and the image resolution was 4068 × 3456 px. The surface illumination was between 1500 and 3000 lx. Each full image was segmented into 256 × 256-px sub-images. Each image represents a physical area of approximately 1000 mm × 850 mm and each sub-image represents a physical area of approximately 60 mm × 60 mm. The authors analyzed the SDNET2018 dataset using the AlexNet DCNN architecture in fully trained (FT) and transfer learning (TL) modes using the computational setup and procedure described by Dorafshan et al. [8]. Benchmarking results, including the sizes of the training and testing datasets, number of epochs required for training, and accuracy of classification of the testing dataset, are presented in Table 2.

**Table 2:** Benchmark for SDNET2018 image classification using AlexNet.

**Image description**	**No. sub-images**		**DCNN mode**	**Training epochs**	**Accuracy (%)**
	***Training***	***Testing***			
Bridge deck	12,259	1,361	FT	32	90.45
			TL	10	91.92
Wall	16,324	1,814	FT	30	87.54
			TL	9	89.31
Pavement	21,900	2,434	FT	30	94.86
			TL	10	95.52
